# Up‐regulation of circular RNA hsa_circ_0037909 promotes essential hypertension

**DOI:** 10.1002/jcla.22853

**Published:** 2019-03-12

**Authors:** Xingjie Bao, Xin He, Shuying Zheng, Jihan Sun, Yizhe Luo, Ronghui Tan, Jinshun Zhao, Fade Zhong, Lina Zhang

**Affiliations:** ^1^ Department of Preventative Medicine, Zhejiang Provincial Key Laboratory of Pathological and Physiological Technology Medicine School of Ningbo University Ningbo China; ^2^ Ningbo Municipal Blood Center Ningbo China

**Keywords:** biomarker, essential hypertension, hsa_circ_0037909, mir‐637

## Abstract

**Aims:**

Essential hypertension (EH) is a high prevalence disease facing a public health challenge. People were little known about the genetics of diagnosing the cause of EH. Circular RNAs that have a continuous cycle of covalent closure, without affected by RNA exonuclease, and are more stable and hard to degrade may involve into the molecule regulation mechanism of EH as an important biomedical.

**Methods:**

qRT‐PCR was used to analyze circRNAs in total volume of human blood and the induced human aortic endothelial cells (HAECs) and human umbilical vein endothelial cells (HUVECs). Our case‐control study was involved with 48 pairs of case controls with sex and age (±3 years) match. We conducted *t* test, Pearson's χ^2^ test, and receiver operating characteristics (ROC) curve analysis for the corresponding analysis.

**Results:**

The expression level of hsa_circ_0037909 in EH patients was significantly higher than that in the healthy controls (*P* = 0.007), and the expression level of hsa‐miR‐637 in EH patients was significantly lower in than that in the healthy controls (*P* = 0.039); the same result appears in the HAECs and HUVECs. Hsa‐miR‐637 (adjusted *P = *0.018), hsa_circ_0037909 (adjusted *P = *0.005), HDL (adjusted *P = *0.024), and serum creatinine (adjusted *P = *0.014) were brought into the model which performed logistic regression analysis. The combination of two RNAs was excellent (*P* < 0.001) through ROC curve analysis. Hsa_circ_0037909 was significantly positively correlated with serum creatinine (*P* < 0.001) and low‐density lipoprotein (LDL) (*P* = 0.017).

**Conclusions:**

Our findings suggested that the combination of hsa_circ_0037911 and hsa‐miR‐637 may be a significant important biomarker for early diagnosis of EH. Hsa_circ_0037909 may affect serum creatinine or LDL leading to the formation of EH.

## 1 INTRODUCTION

Essential hypertension (EH), with age‐related, chronic, and frequent disorder, is prevalent in 40% of adults aged 25 and over as a public health problem. There are many complications of EH, such as hypertensive heart disease, peripheral vascular disease, and retinopathy.^1^ Since EH is a complex multifactorial disease, patients who diagnosed with EH are little known about the etiology. Unraveling the complete etiology to EH is proved to be challenging.

Circular RNAs (circRNAs) have a continuous cycle of covalent closure and without affected by RNA exonuclease.^2^ circRNAs, different from traditional linear RNA, are more stable and not easy to degrade with neither 5' to 3' polarity nor polyadenylated tail.^3^

Functionally, in recent studies, circRNAs are able to act as miRNAs sponges in cells due to the rich binding sites of microRNAs (miRNAs). Thus, the inhibition of miRNA on target was eliminated and the number of target genes was increased. This mechanism is called the competitive endogenous RNA (ceRNA) mechanism.^4^ The RNAs research found that circRNAs play a key role in the development of the disease like EH.

Hsa_circ_0037909 is located in the fragment 11 984 918‐11 990 642 bp on chromosome 16 with 340nt sequence length that included three exons, and the association gene symbol is GSPT1. According to the bioinformatics analysis, it may be associated with EH and become an accurate prediction for it.

In the current study, combination of circBase database and bioinformatics analysis, we tested whether the expression levels of hsa_circ_0037909 were related to EH risk. In addition, the sensitivity of hsa_circ_0037909 analysis was carried out by receiver operating characteristics (ROC) graphs. According to the results, hsa_circ_0037909 may be a potential biomarker for EH as a screening and prevention tools.

## 2 MATERIALS AND METHODS

### 2.1 Study population

In the circRNA microarray step, we collected five newly diagnosed EH and five age‐matched (±3 years) and gender‐matched non‐EH in Yinzhou People's Hospital, and in the second validation phase, we selected 48 pairs of EH and non‐EH. Ages from 30 to 75 were defined for all participants. Cases with stroke, secondary hypertension, renal, diabetes, or metabolic disorders were excluded in this study. According to the 2013 guidelines for management of hypertension, hypertensive heart disease (EH groups) bases on systolic blood pressure (SBP) ≥ 140 mm Hg and/or diastolic blood pressure (DBP) ≥ 90 mm Hg, and non‐hypertensive heart disease (control groups) bases on SBP <120 mm Hg and DBP <80 mm Hg. All research subjects had following 12‐hour overnight fasting, using ethylenediaminetetraacetic acid to gather venous blood and store at −80℃. All participants' written informed consents were obtained before being enrolled in the study.

### 2.2 circRNAs and miRNAs extract and reverse transcription

Total RNAs were extracted from peripheral blood samples using TRIzol kit (TransGen, Beijing, China). An ultramicrospectrophotometer (NanoDrop4002; Thermo Fisher Scientific, Waltham, MA, USA) was used to measure the concentration and purity of extracted RNA. Chemistry‐Immuno Analyzer (Tokyo, Japan) was used to measure biochemical variables. The reverse transcription of circRNAs and miRNAs were carried out by GO ScriptTM Reverse Transcription System (Promega, Beijing, China) and miRcute Plus miRNA First‐Strand cDNA Synthesis Kit (TianGen, Beijing, China), respectively.

### 2.3 Validation of circRNAs and miRNAs expression by qRT‐PCR

According to expression profile analysis,^5^ we selected one circRNA and its corresponding miRNAs to validate. The cDNAs of circRNAs and miRNAs were involved with quantitative real‐time polymerase chain reaction (qRT‐PCR) analyses carried out by GoTaq^®^ Real‐Time PCR Systems reagent kit (Promega) and miRcute Plus miRNA qPCR Detedtion Kit (TianGen), respectively. Following the manufacturer's instruction performed on Roche LightCycler 480 II (Basel, Switzerland), GAPDH is an internal reference for circRNAs, whereas cel‐miR‐39 is an external control for miRNAs.

### 2.4 Cell culture

Human aortic endothelial cells (HAECs) were cultured in RPMI 1640 medium that is 10% of fetal bovine serum. Human umbilical vein endothelial cells (HUVECs) were cultured with Dulbecco's modified Eagle's medium. At 37°C, two cells were included 5% of carbon dioxide. Each orifice cell was divided into experimental and control groups. The control groups were routinely cultured, and the experimental groups were cultured with TNF‐α 1 μmol/L 24 hours, 1 μmol/L 48 hours, 10 μmol/L 24 hours, and 10 μmol/L 48 hours. Then, circRNAs and miRNAs of cells’ extract, reverse transcription, and validation are the same as the population samples. Three samples were tested at a time, and each cell line was repeated at least three times.

### 2.5 Statistical analyses

SPSS 19.0 software package（IBM Corp., Armonk, NY, USA) was used to analyze experimental data. The expression of circRNAs and miRNAs was calculated according to △*C*_t_ = *C*_t_ (circRNAs) − *C*_t_ (GAPDH) or △*C*_t_ = *C*_t_ (miRNAs) − *C*_t_ (cel‐miR‐39). Mean ± SD was appeared by continuous numeric data, and Pearson's *χ* ^2^ test was performed on categorical variables data. Investigating the effect of predictors adjusting the confounders was conducted with multivariable logistic regression (stepwise). Investigating the diagnostic accuracy was measured by the ROC curve analysis. *P* value <0.05 was considered statistically significant.

## 3 RESULTS

### 3.1 Baseline data of study subjects

Our study aimed to found the circRNAs signature to understand the EH mechanisms underlying the pathogenesis in EH patients; 48 pairs of case controls with sex and age (±3 years) match were enrolled from Yinzhou People's Hospital. In Table 1, all participants were within normal ranges in the aspect of baseline data. As expected, there were no statistically significant differences in gender and age. Eosinophilic granulocyte ratio (*t* = 6.92, *P* < 0.001) showed statistically significant differences between two groups.

**Table 1:** Comparison of characteristics between controls and EH group

Characteristics	Controls (mean ± SD)	EH (mean ± SD)	*t/χ* ^2^	*P*
Age (y)	55.31 ± 9.76	55.58 ± 9.67	−0.14	0.892
Sex (M/F)	30/18	30/18	/	/
BMI (kg/m^2^)	23.07 ± 2.68	23.88 ± 2.69	−1.55	0.14
ALT (IU/L)	23.10 ± 14.11	24.34 ± 16.10	0.40	0.69
AST (IU/L)	23.88 ± 6.38	23.98 ± 7.44	0.07	0.94
Creatinine (μmol/L)	78.87 ± 16.52	76.02 ± 21.31	0.73	0.47
TGs (mmol/L)	1.65 ± 0.73	1.45 ± 0.55	−1.52	0.13
CHO (mmol/L)	5.29 ± 0.90	5.18 ± 0.75	−0.67	0.51
HDL (mmol/L)	1.41 ± 0.31	1.31 ± 0.26	−1.75	0.84
LDL (mmol/L)	2.77 ± 0.64	2.88 ± 0.85	−0.69	0.49
Eosinophilic granulocyte ratio	2.34 ± 1.67	1.74 ± 1.09	6.92	**<0.001** *
SBP (mm Hg)	122.27 ± 10.02	140.29 ± 15.01	−6.92	**<0.001** *
DBP (mm Hg)	77.33 ± 7.19	86.94 ± 1.54	−5.21	**<0.001** *
Smoking (Y/N)	7/11	11/7	1.78	0.18
Drinking (Y/N)	6/12	10/8	1.80	0.18
Hsa_circ_0037909	6.31 ± 1.59	5.38 ± 1.67	2.77	**0.007** *
Hsa‐miR‐637	1.33 ± 1.14	1.91 ± 1.51	−2.10	**0.039** *

ALT: alanine transaminase; AST: aspartate aminotransferase; BMI: body mass index; CHO: total cholesterol; DBP: diastolic blood pressure; EH: Essential hypertension; HDL: high‐density lipoprotein; LDL: low‐density lipoprotein; SBP: systolic blood pressure; TGs: triglycerides.

* *P* value ≤0.05 is in bold.

### 3.2 Validation of gene expression in blood samples

**Figure 1:**
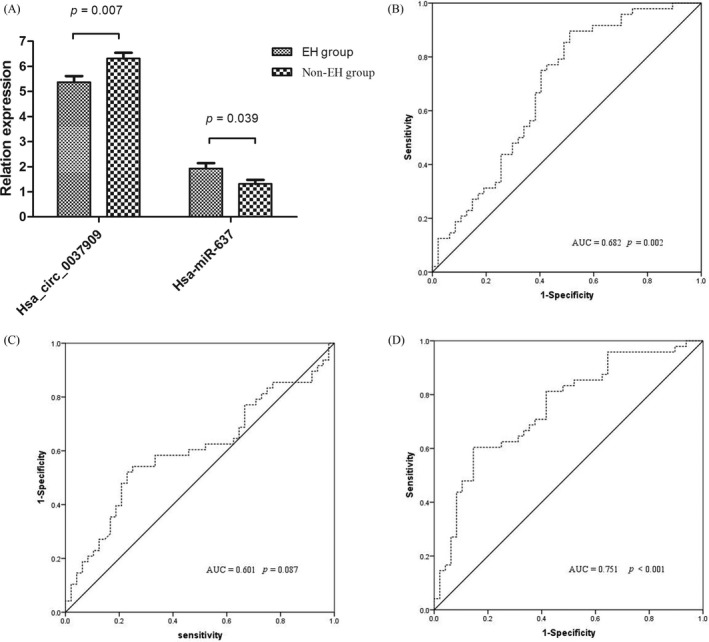
A, Expression of hsa_circ_0037909 and hsa‐miR‐637 detected by RT‐PCR in all cases; B, a receiver operating characteristic (ROC) curve showing hsa_circ_0037909; C, a ROC curve showing hsa‐miR‐637; D, a ROC curve showing combination of hsa_circ_0037909 and hsa‐miR‐637

According to microarray data,^5^ we selected one up‐regulated circRNA and one miRNA that corresponds circRNA. In particular, the GAPDH was considered to be an internal control for normalizing circRNAs and the cel‐miR‐39 was considered to be an external control for normalizing miRNAs.^5^ As shown in Table 1 and Figure 1A, hsa_circ_0037909 was up‐regulated (*P* = 0.007) and hsa‐miR‐637 was down‐regulated (*P* = 0.039) in EH group. Approximately 1.17‐fold increase in hsa_circ_0037909 and 0.70‐fold decrease in hsa‐miR‐637 had observed in EH cases compared to that in other groups, respectively.

### 3.3 Diagnostic accuracy of RNAs

After logistic regression analysis adjusted, we found that some variables were brought into the model including hsa‐miR‐637 (OR = 3.081), hsa_circ_0037909 (OR = 0.193), HDL (OR = 0.004), and serum creatinine (OR = 1.139) in Table 2. Our model showed that down‐regulated hsa‐miR‐637 and up‐regulated hsa_circ_0037909 can be risk factors for EH.

**Table 2:** Effect of predictors for EH

Variable	OR (95%CI)	Wald	*P *value
Sex	5.094 (0.188, 137.9)	0.936	0.333
Age (y)	0.959 (0.838, 1.098)	0.368	0.544
ALT (IU/L)	0.934 (0.799, 1.092)	0.732	0.392
AST (IU/L)	1.055 (0.768, 1.448)	0.109	0.741
Scr (μmol/L)	1.139 (1.026, 1.264)	5.987	**0.014** *
Glu (mmol/L)	0.021 (0.218, 5.827)	0.021	0.886
PDW (mmol/L)	0.109 (0.018, 0.665)	5.778	**0.016** *
BUN (mmol/L)	0.871 (0.389, 1.954)	0.112	0.738
WBC count	0.992 (0.452, 2.174)	0.000	0.984
TC (mmol/L)	17.968 (0.606, 533.076)	2.789	0.095
LDL (mmol/L)	0.133 (0.007, 2.520)	1.807	0.179
HDL (mmol/L)	0.004 (0.000, 0.484)	5.086	**0.024** *
TGs (mmol/L)	0.048 (0.004, 0.593)	5.600	**0.018** *
BMI (kg/m^2^)	1.143 (0.780, 1.675)	0.470	0.493
Smoking	0.001 (0.000, 0.678)	4.319	**0.038** *
Drinking	683.1 (0.854, 546117.3)	3.663	0.056
Hsa‐miR‐637	3.081 (1.217, 7.799)	5.637	**0.018** *
Hsa_circ_0037909	0.193 (0.061, 0.616)	7.716	**0.005** *

ALT: alanine transaminase; AST: aspartate aminotransferase; BMI: body mass index; BUN: blood urea nitrogen; EH: Essential hypertension; Glu: glucose; HDL: high‐density lipoprotein; LDL: low‐density lipoprotein; PWD: mean platelet distribution width; Scr: serum creatinine; TC: total cholesterol; TGs: triglycerides.

* *P* value ≤0.05 is in bold.

In addition, independent hsa_circ_0037909 (Figure 1B; AUC = 0.682) or hsa‐miR‐637 (Figure 1C; AUC = 0.601) may not be an ideal diagnostic marker through ROC curve analysis, whereas the combination of two RNAs was excellent (Figure 1D; AUC = 0.751).

**Figure 2:**
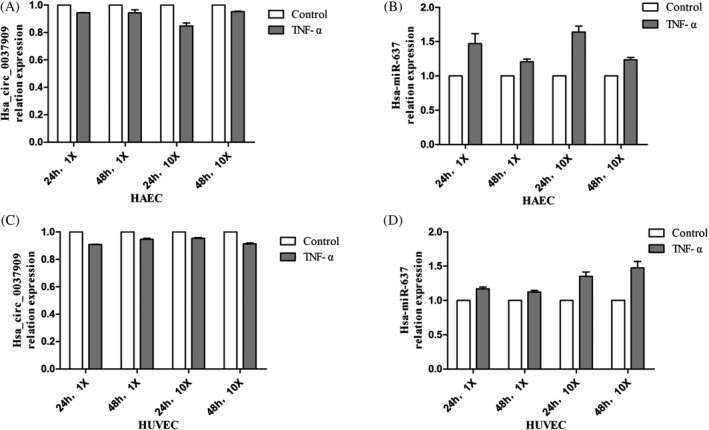
A, Correlation of hsa_circ_0037909 and Scr; B, correlation of hsa_circ_0037909 and low‐density lipoprotein

Further data analysis showed that hsa_circ_0037909 has a significant positive correlation with serum creatinine (Figure 2A; *r* = 0.396) and low‐density lipoprotein (LDL) (Figure 2B; *r* = 0.246, *P* = 0.017).

### 3.4 Effect of gene expression in endothelial cells

**Figure 3:**
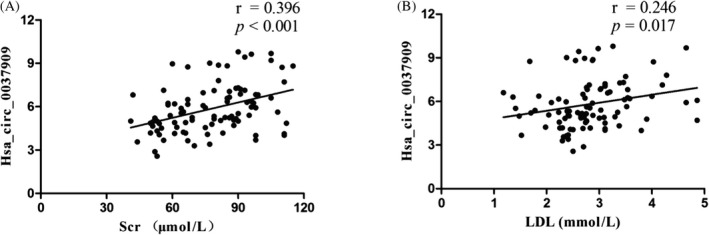
A, Expression of hsa_circ_0037909 detected by RT‐PCR in human aortic endothelial cells (HAECs); B, expression of hsa‐miR‐637 detected by RT‐PCR in HAECs; C, expression of hsa_circ_0037909 detected by RT‐PCR in human umbilical vein endothelial cells (HUVECs); D, expression of hsa‐miR‐637 detected by RT‐PCR in HUVECs

Since endothelial cells are related to the pathology of target tissue damage of hypertension, we used TNF‐α to induce the formation of EH mimics and examined changes about hsa_circ_0037909 and hsa‐miR‐637. We detected HAECs and HUVECs, the predicted target gene expression in both experimental and negative control groups. qRT‐PCR analysis showed that two kinds of endothelial cells significantly up‐regulated hsa_circ_0037909 expression levels after treatment with TNF‐α (Figure 3), and hsa‐miR‐637 was significantly lower. In addition, the degree of up‐ or down‐regulation increases, as the treatment concentration and time increase. Taken together, above results suggested that hsa_circ_0037909 and hsa‐miR‐637 may play roles in endothelial cell injury.

## 4 DISCUSSION

CircRNAs in great part are derived from exons, and a few RNAs are formed by direct cyclization of introns.^6^

Increasing circRNAs has been found in a variety of species over time, including humans.^7^ The increasing molecular functional circRNAs were revealed.^8^ With the characteristic of back‐splicing reaction, circRNAs have been inhibited through ADAR and DHX9, and facilitated by exon skipping, specific protein factors, or reverse complementary sequences.^9^ Furthermore, depending on its subcellular localization, circRNAs might play different roles; for example, RNA polymerase II can be recruited by most circRNAs to regulate the expression of specific genes.^10^ Also, circRNAs serve as sponges for miRNAs and cytoplasmic proteins, or at transcriptional, post‐transcriptional, and translational to regulate gene expression levels.^11^ Recent studies have shown that circRNAs serve as biomarkers or therapeutic targets for tumor diagnosis since playing great roles in various tumor biological processes.^12^ Importantly, circRNAs involved in the pathogenesis of cardiovascular disease have been revealed, for example, circRNA as miRNA‐223 sponges to control cardiac hypertrophy and heart failure.^13^ Overall, circRNAs have great potential for disease progression. Nevertheless, research of circRNAs in multifactorial chronic diseases, especially EH, has been restricted. Besides its action mechanism and clinical application value are unclear yet.

According to the results of microarray, we found that hsa_circ_0037909 regulated hsa‐miR‐637, and hsa_circ_0037909 was up‐regulated and hsa‐miR‐637 was down‐regulated. In validation step, the finding was consistent with microarray (Figures 1 and 3). This is the first time that hsa_circ_0037909 acted as higher regulated and hsa‐miR‐637 acted as lower regulated in the whole blood and endothelial cells simultaneously.

Hsa‐miR‐637 levels decrease with tumor that highly correlated. It could act as a suppressor regulated on follicular thyroid carcinomas,^13^ ovarian cancer,^14^ and hepatocellular carcinoma,^15^ and it also participated in the differentiation of cells, such as human mesenchymal stem cells,^15^ migration and invasion of glioma cell, and human pancreatic ductal adenocarcinoma cells via suppressing Akt1 expression.^16^ At the same time, hsa‐miR‐637 can performed as a marker of follicular thyroid cancer,^17^ even hypertension.^18^ In the ATP6V0A1 polymorphism, the miR‐637 expression has been associated with EH development.^19^ In another study, down‐regulation of hsa‐miR‐637, which regulates the CDK6 expression from lung smooth muscle cells, increases the risk of hypoxia‐induced pulmonary hypertension.^20^ A team of O'Connor et al^21^ DT found that hsa‐miR‐637 level was decreased in the plasma of hypertension patients. Some studies have shown that known genes have been regulated by hsa‐miR‐637, for example, RDH11, KLHL7, CERKL, AIPL1, and USH1G.^22^ In conclusion, we have enough reasons to speculate that hsa‐miR‐637 may affect EH occurrence.

Some scientists discovered that hsa_circ_0003575 was a higher expression in HUVECs, which induced by oxidative modified LDL (oxLDL), and promoted the HUVECs proliferation and angiogenesis.^23^ The mechanism is explained by the circRNAs, as a miRNAs sponge, with an indeterminate amount of miRNA response elements in its cyclic structure. Therefore, circRNAs may absorb the number of miRNAs and promote the expression of mRNAs.^24^ In the current study, we discovered that hsa_circ_0037909 was up‐regulated not only in whole blood, but also in both HAECs and HUVECs. The corresponding hsa‐miR‐637 is down‐regulated. Meanwhile, there is a positive correlation between hsa_circ_0037909 and LDL. The relationship between serum creatinine and hypertension has been released.^25^ Luan et al also found that circRNAs have a positive correlation with serum creatinine and act as a sponge for hsa‐miR‐150.^26^ Our results have shown the same tendency in hsa_circ_0037909 expression levels and serum creatinine concentration. Taken together, we have enough reason to prove that the combination of hsa_circ_0037909 and hsa‐miR‐637 will affect biochemical indicators level and endothelial cells function.

Numerous studies have been reported that different circRNAs expression involved in the pathogenesis of cardiovascular system disease. Chen et al used RT‐PCR to confirm that hsa_circ_0003573 is up‐regulated in HUVECs.^23^ CircANRIL is transcribed on chromosome 9p21 at the atherosclerosis‐related position, induces apoptosis, and then inhibits the macrophages of smooth muscle cells and proliferation.^27^ Large quantity circRNAs were distinguished in hypoxia‐induced endothelial cells, for example, cDENND4C. One experiment demonstrated that cZNF292 reticent inhibits spheroid germination and endothelial cell tube formation.^28^ CircRNA_000203 sponge of miR‐26b‐5p, Col1a2, Col3a1, and α‐SMA increases expression and then stimulates pro‐fibrosis circRNA in mouse cardiac fibroblasts.^29^ Moreover, profiling and bioinformatics analyses indicated that circRNA is a risk factor for hypertension.^30^

Taken together, this result presents that hsa_circ_0037909 was significantly increased and hsa‐miR‐637 was significantly decreased in the EH group and TNF‐α‐induced HAECs and HUVECs. Their expressions are interrelated with LDL and serum creatinine concentrations. Also, hsa_circ_0037909 may play a meaningful part about EH pathogenesis, which has been suggested. Next, we will build circRNA‐miRNA‐mRNA network models and seek circRNAs functional mechanism with more depth.

## AUTHORS CONTRIBUTIONS

XH, SZ, and JS conceived and designed the experiments. YL, RT, JZ, JZ, and FZ performed the experiments. XB, YL, RT, and LZ analyzed the data and wrote the manuscript.
